# Adenofibroma in a Young Patient: A Rare Entity in an Uncommon Age

**DOI:** 10.1155/2013/308464

**Published:** 2013-12-28

**Authors:** R. Maciel, S. Carvalho, M. Teixeira, M. J. Areias

**Affiliations:** Department of Obstetrics and Gynecology, Centro Hospitalar do Porto, Portugal

## Abstract

Adenofibroma is an extremely uncommon benign tumor composed of glandular and fibrous tissues. It occurs more often in the endometrium but it can also occur in the cervix and extrauterine sites. We report a case of a 32-year-old asymptomatic woman with cervical adenofibroma, first detected in a routine endovaginal ultrasound, as a cervical mass containing multiple cystic components. Histopathologic findings diagnosed its nature. As adenofibromas are very rare, we present this case with a brief review of the literature.

## 1. Introduction

Endometrial adenofibroma is a rare benign mullerian mixed tumor composed of benign epithelial and mesenchymal components. The major proportion of adenofibromas arise from the endometrium (90%); less often occur in the cervix [1] and in other anatomical locations. When this tumor arises in the cervix, women can present with abnormal vaginal bleeding, as a first sign. It can occur in any age but is most commonly seen in peri- or postmenopausal women [2]. There are no typical preoperative patterns (clinical or sonographic) that strongly suggest this diagnosis. The major concern before this benign mixed mesodermal tumor must be its differential diagnosis with other malignant lesions of the uterus, particularly adenosarcoma. Other differential diagnoses are adenomyoma and carcinosarcoma [3, 4].

We report a case of an adenofibroma of the uterine cervix in a young patient.

## 2. Case Report

**Figure 1:**
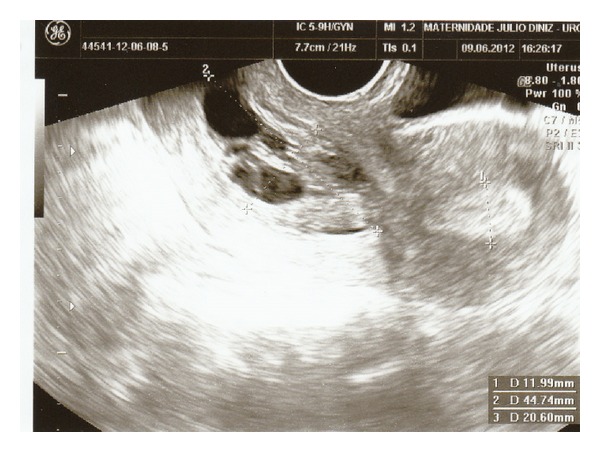
Endovaginal ultrasound: intracervical multicystic mass, with 45 mm in its maximum diameter.

**Figure 2:**
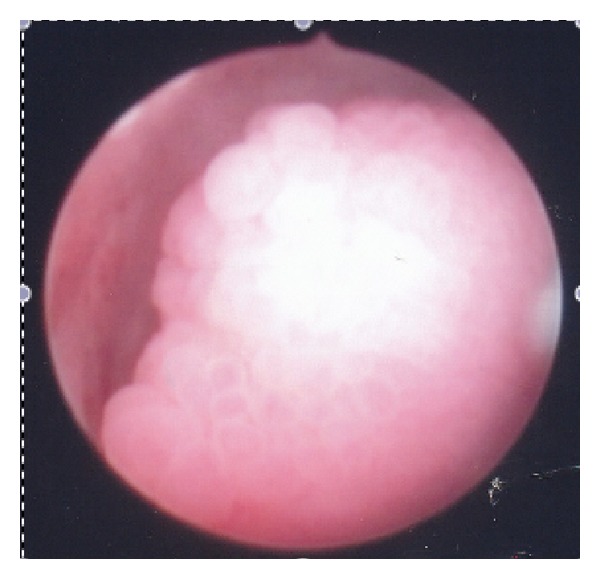
Diagnostic hysteroscopy: firm and pallid polyploidy mass containing multiple cystic spaces and some foci of hemorrhage.

**Figure 3:**
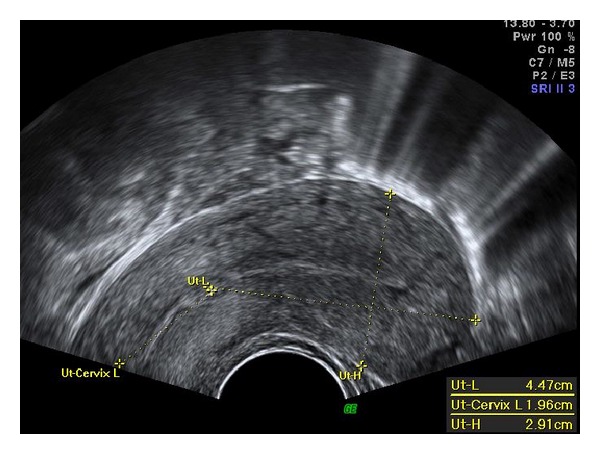
Follow-up endovaginal ultrasound, 3 months later: linear endocervical epithelium.

A 32-year-old, nulliparous, woman went to her gynaecologist for routine procedures. She had no complaints and referred regular menstrual cycles, with oral combined contraception. Pelvic examination was normal. Endovaginal ultrasound revealed an intracervical multicystic mass, with 45 mm in its maximum diameter (Figure 1). Diagnostic hysteroscopy was performed and a firm and palid polypoid mass containing multiple cystic spaces and some foci of hemorrhage was detected (Figure 2). The histopathological examination of the biopsy specimen disclosed an adenofibroma of the uterine cervix: endometrial glands without architectural complexity or cytologic atypia, surrounded by stroma of smooth muscle. An operative hysteroscopy (resectoscopy) with total resection of the tumor was performed. The histological examination confirmed its benign origin. The follow-up ultrasound, three months later, was normal, with a linear endocervical epithelium (Figure 3) and the patient was asymptomatic.

## 3. Discussion

It was in 1971, by Abell, that adenofibroma of the uterine cervix was first described [5]. There have been few reports since then. A literature review carried out using the Pubmed search, with the words “cervical adenofibroma”—and subsequent filters, demonstrated less than 10 manuscripts published.

In fact, as a result of its low incidence, the origin of this tumor is still a source of debate; some authors believe it represents a form of endometriosis with extreme smooth muscle metaplasia—endomyometriosis [6]. More recently, Chu et al. (2012) reported a case of an adenofibroma of the uterine cervix coexistent with endometriosis [3].

Physical examination can be either normal, as the patient presented herein, or it can reveal an enlarged uterus with a polypoid tumor projecting from the cervix.

The sonographic pattern produced by this tumor is an echogenic intracavitary mass with heterogeneous multicystic components, with well-defined margins and low-resistance blood flow. Adenofibroma must be differentiated from endometrial polyp, hyperplasia, and carcinoma. Macroscopically, adenofibroma presents as a broad-based polypoid lesion; commonly has a fibrous consistency and contains dilated cystic spaces. Its size ranges from 2 to 20 cm, with an average diameter of 7 cm [7]. As in other pathologies, histological examination is required to reveal the true nature of the tumor. The diagnosis of an adenofibroma should be considered in the absence of features present in an adenosarcoma such as stromal atypia, periglandular cuffing, and mitotic activity. Polyps differ from adenofibroma in that they have a smoother, rounder mucosal surface that lacks the papillary processes of an adenofibroma; they also have more glands with less cellular and more collagenous stroma. In adenofibromas the central vasculature characteristic of polyps is not found. In case of a stromal mitotic count of more than 1 mitosis per 10 high power fields, marked stromal hypercellularity with periglandular cuffing and/or high stromal atypia, a diagnosis of a low grade adenossarcoma should be made. Authors suggest that many tumors diagnosed as adenofibromas are in fact low grade adenossarcomas [7].

In adenofibroma, the stromal component is by definition morphologically benign. However, it is described the possibility of this tumor to invade the myometrium and the pelvic veins, to recur or even metastasize [3, 8, 9]. We think this can be explained by the almost superimposed pathological findings and eventual missed malignant features. As such, it is understandable that it is described that hysterectomy is the preferred treatment for endometrial adenofibroma; it assures complete excision and it also allows thorough sampling, needed to exclude an adenosarcoma. However, we should emphasize that in almost all the cases reported, the fertile age of these patients was in peri or postmenopause. By this reason, we consider that wide local excision via operative hysteroscopy can be an alternative option to hysterectomy, provided that the completeness of excision is verified and the long term followup available. This is plausible in young women who desire offspring, as in the present case.

In conclusion, although rare, adenofibromas should be considered in the differential diagnosis of a patient with a cervical mass and abnormal vaginal bleeding without clinical evidence of malignancy. Detailed histopathological study is required to differentiate adenofibromas from adenosarcoma.

## References

[1] Haberal A, Cil AP, Gunes M, Cavusoglu D (2005). Papillary adenofibroma of the cervix: a case report. *Ultrasound in Obstetrics and Gynecology*.

[2] Ishiko O, Sumi T, Ueda K, Kawamura N, Ogita S (2002). Uterine cervical adenofibroma associated with Turner’s syndrome in a young woman. *Archives of Gynecology and Obstetrics*.

[3] Chu IL, Chen CL, Hsu CS (2012). Adenofibroma of the uterine cervix coexistent with endometriosis. *Taiwanese Journal of Obstetrics and Gynecology*.

[4] Tahlan A, Nanda A, Mohan H (2006). Uterine adenomyoma: a clinicopathologic review of 26 cases and a review of the literature. *International Journal of Gynecological Pathology*.

[5] Abell MR (1971). Papillary adenofibroma of the uterine cervix. *American Journal of Obstetrics and Gynecology*.

[6] Clement PB (2007). The pathology of endometriosis: a survey of the many faces of a common disease emphasizing diagnostic pitfalls and unusual and newly appreciated aspects. *Advances in Anatomic Pathology*.

[7] Bettaieb I, Mekni A, Bellil K (2007). Endometrial adenofibroma: a rare entity. *Archives of Gynecology and Obstetrics*.

[8] Vellios F, Ng AB, Reagan JW (1973). Papillary adenofibroma of the uterus: a benign mesodermal mixed tumor of Müllerian origin. *American Journal of Clinical Pathology*.

[9] Clement PB, Scully RE (1990). Müllerian adenofibroma of the uterus with invasion of myometrium and pelvic veins. *International Journal of Gynecological Pathology*.

